# Executive Dysfunctions: The Role in Attention Deficit Hyperactivity and Post-traumatic Stress Neuropsychiatric Disorders

**DOI:** 10.3389/fpsyg.2016.01230

**Published:** 2016-08-23

**Authors:** Lía Martínez, Edward Prada, Corina Satler, Maria C. H. Tavares, Carlos Tomaz

**Affiliations:** ^1^Laboratory of Neurosciences and Behavior, Department of Physiological Sciences, University of BrasiliaBrasilia, Brazil; ^2^Faculty of Psychology, Social Sciences Department, Universidad Pontificia Bolivariana Seccional BucaramangaBucaramanga, Colombia; ^3^Faculty of Ceilandia, University of BrasiliaBrasilia, Brazil; ^4^Neuroscience Research Program, University CEUMASão Luis, Brazil

**Keywords:** ADHD, executive functions, inhibitory control, neuropsychiatric disorders, PTSD

## Abstract

Executive functions (EFs) is an umbrella term for various cognitive processes controlled by a complex neural activity, which allow the production of different types of behaviors seeking to achieve specific objectives, one of them being inhibitory control. There is a wide consensus that clinical and behavioral alterations associated with EF, such as inhibitory control, are present in various neuropsychiatric disorders. This paper reviews the research literature on the relationship between executive dysfunction, frontal-subcortical neural circuit changes, and the psychopathological processes associated with attention deficit hyperactivity disorder (ADHD) and post-traumatic stress disorder (PTSD). A revision on the role of frontal-subcortical neural circuits and their presumable abnormal functioning and the high frequency of neuropsychiatric symptoms could explain the difficulties with putting effector mechanisms into action, giving individuals the necessary tools to act efficiently in their environment. Although, neuronal substrate data about ADHD and PTSD has been reported in the literature, it is isolated. Therefore, this review highlights the overlapping of neural substrates in the symptomatology of ADHD and PTSD disorders concerning EFs, especially in the inhibitory component. Thus, the changes related to impaired EF that accompany disorders like ADHD and PTSD could be explained by disturbances that have a direct or indirect impact on the functioning of these loops. Initially, the theoretical model of EF according to current neuropsychology will be presented, focusing on the inhibitory component. In a second stage, this component will be analyzed for each of the disorders of interest, considering the clinical aspects, the etiology and the neurobiological basis. Additionally, commonalities between the two neuropsychiatric conditions will be taken into consideration from the perspectives of cognitive and emotional inhibition. Finally, the implications and future prospects for research and interventions in the area will be outlined, with the intention of contributing scientific reference information that encompasses the knowledge and understanding of executive dysfunction and its relationship with these treated disorders.

## Introduction

### General Aspects of Executive Functions

Executive functions (EFs), also known as executive control or cognitive control, are a complex set of interrelated cognitive processes controlled by its top–down nature (Diamond, 2013) and mediated by dynamic behaviors in order to achieve goal-oriented behaviors that are essential for an individual to solve problems, resist interference, hold attention, learn, make decisions, plan, and regulate their behavior (including in new situations), among other skills necessary for everyday life (Diamond, 2013; Chung et al., 2014).

In the historical background, it has been found that although the term “EFs” was coined by Lezak, it was Luria – his direct predecessor – who first described it, even without mentioning it directly. He proposed a brain functioning model based on clinical findings, which consists of three functional units: (1) motivation arousal (reticular and limbic systems); (2) acquisition, processing, and storage of information (post-rolandic cortical areas); and (3) programming, monitoring, and verification of behavior [dependent on the activity of the prefrontal cortex (PFC); Ardila, 2008]. This complex brain system would be mediated by neuroanatomical and functional hierarchical regions, and would work together to regulate all behavior and mental processes (Jurado and Rosselli, 2007).

Regarding the classification of EFs, inhibition can be highlighted (inhibitory control, including self – behavioral inhibition – and control of interference, selective attention and cognitive inhibition), as well as working memory (WM), cognitive flexibility (also known as mental flexibility; Miyake et al., 2000), reasoning, problem solving, and planning (Collins and Koechlin, 2012; Lunt et al., 2012).

Fuster (2008) was one of the most recognized in the EFs study and was the one who proposed a general theory of the PFC and ideas about the importance of this area in the temporal structure of behavior. He suggested that EFs are cognitive skills that enable the organization of a sequence of actions in pursuit of a goal, and proposed the following cognitive skills as part of EFs: attention (alert, set, spatial attention, sustained attention, and control interference), memory, WM, planning, temporal integration, decision making, and inhibitory control.

**FIGURE 1:**
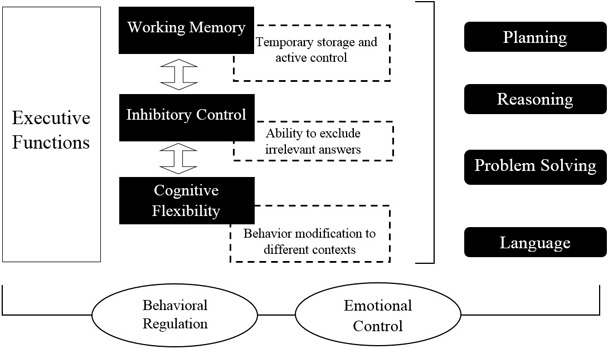
**Main components and characteristics of the EFs.** Source: made by myself.

Recently, there has been a consensus that three basic nuclei compose EFs: WM, cognitive flexibility, and inhibition of prepotent impulses (Hofmann et al., 2012; Diamond, 2013). WM refers to temporary maintenance and active control of information, avoiding distraction (Kane et al., 2001). For its part, cognitive flexibility is the ability to change and modify the course of thoughts or actions to multiple tasks that require it (Elliott, 2003), and inhibition is the ability to inhibit dominant or automatic responses when is necessary (Miyake et al., 2000). See **Figure 1**.

The focus of this study(revision) will be inhibitory control in EFs, describing and expanding its general characteristics, and the neuropsychiatric conditions of attention deficit hyperactivity disorder (ADHD) and post-traumatic stress disorder (PTSD), with special emphasis on cognitive and emotional perspectives. This paper reviews the research literature on the relationship between executive dysfunction, frontal-subcortical neural circuit changes, and psychopathological processes associated with ADHD and PTSD.

### Neuroanatomy of Executive Functions

Executive Functions are closely linked to the activity of the frontal lobes, which establish a significant correlation on a clinical-anatomical level regarding clear evidence from case reports of patients with difficulties that arise after brain injuries to frontal areas, and which result in alterations called “executive dysfunction” or “frontal lobe dysfunction” (Elliott, 2003). But recently, with the advance of technology, various imaging methods [such as magnetic resonance imaging (MRI), functional magnetic resonance imaging (fMRI), and Positron emission tomography (PET)] there is evidence that executive functioning is associated with several distributed networks (Chung et al., 2014), which include not only the frontal regions of the cortex but the subcortical areas too (Collette et al., 2006; Bonelli and Cummings, 2007; Jurado and Rosselli, 2007; Marvel and Desmond, 2010).

**FIGURE 2:**
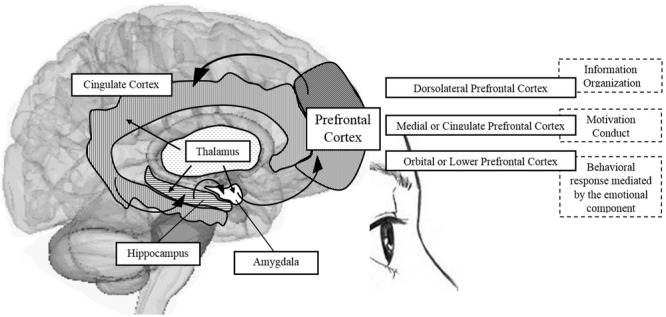
**Schematic representation of brain areas associated with the PFC and participation in shaping some components of EFs.** Source: made by myself.

The frontal lobe is anatomically shaped, in both human and non-human primates, by the dorsolateral PFC, the medial and cingulate PFC, and the orbital PFC (Fuster, 2002). These areas are involved in complex cognitive processes; the dorsolateral region in WM, the anterior and medial areas in desire and motivation, and the orbital region in inhibitory impulse control and interference (Fuster, 2002). See **Figure 2**.

Some authors have emphasized the “circuits,” specifically the frontal-subcortical circuits, claiming that the dorsolateral prefrontal circuit is involved in the organization of information to facilitate a response; the anterior cingulate circuit is related to the conduct of motivation; and orbitofrontal circuit translates the limbic and emotional information into behavioral responses. For these authors, the deterioration of EFs resulting in apathy and impulsivity, for example, is a sign of dysfunction of the frontal-subcortical circuit (Bonelli and Cummings, 2007). Additionally, it is important to mention that the EFs, although closely linked with the PFC, do not rely only on this structure, but also on the intact functioning of the cortical-striatal dopaminergic system (Elliott, 2003). It is the neurobiological basis of EFs, occupying a favored place for “orchestrating all of these functions,” as it has the ability to receive and send information to almost all sensory and motor brain systems (Arnsten and Li, 2005).

Bonelli and Cummings (2007) reported that neuropsychiatric expressions associated with neurodegenerative diseases are closely linked to impairments in neurocircuits generated from frontal connections with other brain areas, especially subcortical areas, since the effector mechanisms allowing the organism to act efficiently within its environment are disturbed. This supports the link that has been found between faiths and different psychopathological and behavioral disorders (Närhi et al., 2010; Biederman et al., 2011).

According to Jurado and Rosselli (2007) there are numerous neurodevelopmental and adult disorders for which alterations to the EFs have been identified, linked to the symptoms also found in people with damaged frontal lobes, such as sustained and selective attention, impulsivity, hyperactivity, and deficits in warning systems, WM, the three mechanisms of inhibitory control (waiting, impulse, or interference control) and self-regulation of behavior, as well as perseveration, cognitive rigidity and planning difficulties, among others.

### Inhibitory Control

Inhibitory control is considered an important EF due to its ability to control attention, behavior, thoughts and/or emotions, in order to override a strong internal or external predisposition to distraction, and to do so whenever appropriate or necessary according to the demands of the environment. The term “inhibition” refers to several different types of inhibition, such as response or motor inhibition, cognitive inhibition, interference control, motivational inhibition, and the automatic inhibition of attention (Diamond, 2013).

Inhibitory control refers to the mental processes responsible for intentional and voluntary control, involving the ability to resist interferences from irrelevant information and to erase previously relevant information that may create incentives in the short term, but that are not functional to solving a particular problem (Carlson and Wang, 2007). Motor inhibition is defined as the individual’s ability to inhibit a behavioral response to a stimulus, i.e., the ability to inhibit a strong behavioral tendency (Diamond, 2006). On the other hand, cognitive inhibition refers to the suppression of irrelevant information in WM (Miyake et al., 2000). It has been found that failures in cognitive inhibition are associated with internalizing problems; on the contrary, the failures are related to behavioral inhibition outsourcing the problems and are based on the individual’s ability to inhibit the behavioral response to a stimulus, specifically in tasks that require setting a plan and eliminating wrong answers (Diamond, 2013).

The existence of multiple inhibition types suggests the probability of an overlap between the different brain regions that underlie these inhibitory processes (Collette et al., 2001).

It is argued that the neural substrate of inhibitory control lies mainly in dorsolateral, orbitofrontal and ventromedial areas of the PFC (Fuster, 2002; Ditye et al., 2012), associated with the right prefrontal gyrus (Ditye et al., 2012). Thus, interactions between the right PFC, the basal ganglia, the primary motor regions and the medial temporal lobe are important for the expression of inhibitory control (Aron et al., 2004).

Neuroimaging studies that address inhibition processes have shown the involvement of several regions in the cingulate, prefrontal, parietal, and temporal areas (Collette et al., 2001). However, the exact role of the regions associated with inhibition processes is not completely defined, because the regions mentioned are quite heterogeneous (Collette et al., 2006). Some neuropsychiatric disorders are closely linked to defects in frontal-subcortical neurocircuits. These circuits participate in a variety of neuropsychiatric disorders such as Tourette’s syndrome, Huntington’s disease, obsessive-compulsive disorder, ADHD, schizophrenia and mood disorders, which occur as a result of changes in the orbitofrontal circuit and therefore in inhibitory control (Bonelli and Cummings, 2007).

## Attention Deficit Disorder/Hyperactivity

### Characteristics, Prevalence, Diagnostic Criteria, and Comorbidity

Attention deficit hyperactivity disorder is one of the most common problems that develop during neurodevelopmental childhood, considered a disturbance and prevalent in approximately 5% of children and 2.5% of adults (American Psychiatric Association [APA], 2013).

According to several studies, it is estimated that up to 67% of these children will ontinue to exhibit symptoms in adulthood (Lundervold et al., 2011; Ranby et al., 2012). However, the prevalence data reported in the literature varies due to the range of diagnostic criteria and assessment methods used in the studies. With regard to symptoms, Sobanski et al. (2010) have postulated that hyperactivity behavior tends to decrease with age, while inattention increases.

Adolescents and adults with ADHD have a higher risk of failure in school, emotional problems, difficulties in social relationships and sometimes trouble with the law.

The American Psychiatric Association [APA] (2013) argues that many parents first observe excessive motor activity when the child is in early childhood, with symptoms that are difficult to distinguish from normal behavior, which can be variables before 4 years of age. Furthermore, ADHD is often identified at school when inattention becomes more pronounced and affects performance, stabilizing during early adolescence, but possibly worsening with the onset of antisocial behavior. Even so, for most individuals with ADHD the symptoms of motor hyperactivity become less noticeable during adolescence or adulthood, but difficulties associated with restlessness, inattention, planning and impulsivity may persist.

According to the American Psychiatric Association [APA] (2013) in its Diagnostic and Statistical Manual of Mental Disorders (DSM-5), ADHD is defined as a persistent pattern of behavior that manifests itself in various contexts (school/work, home, social, etc.) that interferes significantly with the development and functioning of the individual with the disorder. Thus, the APA states that the symptoms fall into two major predominant areas: inattention, and hyperactivity and impulsivity. Inattention manifests behaviorally with deviations at work, lack of persistence, difficulty maintaining attention, and disorganization, which are not due to a challenge or a lack of understanding; hyperactivity is reflected in excessive motor activity when it is not adequate; and impulsivity is evidenced by hasty actions that are taken without reflection, resulting from a desire for immediate reward or an inability to delay gratification. These characteristics are reflected in the established diagnostic criteria. See **Table 1**.

**Table 1:** Summary of criteria and specific symptoms for the diagnosis of ADHD (American Psychiatric Association [APA], 2013).

Inattention	Hyperactivity/impulsivity
(1) Failure to pay attention to details or careless mistakes are made in schoolwork, work, or during other activities.	(1) Fidgets, claps hands or feet, or squirms in seat.
(2) Difficulty sustaining attention in tasks or recreational activities.	(2) Standing up in situations where they are expected to remain seated.
(3) Does not seem to listen when spoken to directly.	(3) Runs around or climbs in situations where it is not appropriate (may be limited to fidgeting in adolescents or adults).
(4) Does not follow instructions and fails to finish schoolwork, chores, or work duties.	(4) Is unable to play or engage in leisure activities quietly.
(5) Difficulty organizing tasks and activities	(5) Is “busy,” acting as if impelled by a “motor.”
(6) Avoids, dislikes, or lacks interest in starting tasks that require sustained mental effort.	(6) Talks excessively.
(7) Loses things necessary for tasks or activities.	(7) Responds unexpectedly or before a question has been concluded.
(8) Is easily distracted by external stimuli.	(8) Has difficulty taking turns.
(9) Forgets everyday activities.	(9) Disrupts or interferes with others (adolescents and adults may interfere or get ahead of what others are doing).
**Combined**	
Criteria for inattention (6+ symptoms) and hyperactivity-impulsivity (6+ symptoms) are met.	

*The set of ADHD symptoms usually manifests before 12 years of age, presenting six or more of them (in older adolescents and adults aged 17 years of age, at least five symptoms are required) to diagnose any subtype of ADHD, maintained for a period exceeding 6 months, with an intensity that is incompatible with the level of development and has a direct negative impact on the social and academic/work activities of the individual.*

Regarding the diagnosis of ADHD, Arnett et al. (2013) believe that it must be mainly clinical, as there is no additional examination or neurobiological sign itself that is final; so it must be established based on clinical symptoms. Confirmation via questionnaires, neuropsychological tests and neuropsychological studies is needed for these patients, especially if they are validated by specific neuropsychological batteries that allow reassessments to control the patient’s development regarding the indicated treatment.

It has been found that according to clinical research and the discussion in the DSM-5 (American Psychiatric Association [APA], 2013), comorbid disorders are common in individuals with ADHD. For example, oppositional defiant disorder coincides with ADHD in about half of children with combined prevalence and in about a quarter of predominantly inattentive children and adolescents; conduct disorder occurs in about a quarter of children and adolescents with combined presentation, depending on age and context; a lower percentage of children with ADHD have symptoms that meet criteria for disruptive mood dysregulation disorder; a specific learning disorder commonly occurs along with ADHD; anxiety disorders and major depressive disorders occur in a minority of individuals with ADHD, but more often than in the general population; intermittent explosive disorder occurs in a minority of adults with ADHD, but with levels above the general population; substance abuse disorders are present only in a minority of adults with ADHD, yet they are relatively more frequent among adults with ADHD than in the general population; and antisocial personality disorder and other personality disorders may be associated with ADHD in adults. Other disorders that may be comorbid with ADHD are obsessive-compulsive disorder, tic disorder and the autistic spectrum.

### Cognitive Impairment in Attention Deficit Disorder/Hyperactivity

Attention deficit hyperactivity disorder has important cognitive consequences, especially in relation to EFs. Additionally, it is known that the disorder is not characterized by cognitive difficulties alone, but is also marked by an altered emotional level (recognition, regulation, and expression of emotions; Sobanski et al., 2010), a product of dysfunction in executive control processes; according to the DSM-5 (American Psychiatric Association [APA], 2013), this can generate a long-term impact on the operation and development of the individual.

The implications of ADHD, as well as being analyzed through measurements with neuropsychological and cognitive tests, is also studied using various neurological, neurophysiological and neuroimaging tests, with the electroencephalogram (EEG) having demonstrated its effectiveness. Recently, research related to the measurement of brain activity in individuals with ADHD showed a difference in activity patterns between children with and without ADHD, by evaluating electrophysiological variables such as reaction time (RT) in cognitive tasks (McLoughlin et al., 2014), in inhibitory control tasks (Bruckmann et al., 2012) and exposure to emotional stimuli (Singhal et al., 2012).

In general, the literature shows the existence of cognitive deficits in individuals with ADHD, such as deficits in attention, EFs, memory, and perception (Arnett et al., 2013). Thus, the deficit of individuals with ADHD, originated in dysfunction of the frontal lobes, specifically in the PFC, leading primarily to a delay or reduction of EFs, a basic prerequisite for the successful development of a variety of cognitive processes for achieving goals (Funahashi and Andreau, 2013).

A result of the alterations in the PFC, individuals with ADHD often have a syndrome associated with executive control disorders, mainly manifesting inhibitory difficulties in WM and planning (Willcutt et al., 2005), as well as productivity and creativity, and inability to abstract ideas and to anticipate the consequences of behavior, leading to increased impulsivity (Arnett et al., 2013); on the other hand, they also have significant difficulties modulating affective states and recognizing and understanding emotional information, which results in elevated levels of aggressiveness, irritability, or frustration (Martel and Nigg, 2006).

### Neurobiology of Attention Deficit Disorder/Hyperactivity

Scientific studies demonstrate the involvement in ADHD of several neuroanatomical structures within the frontal cortex, especially the prefrontal area, the basal ganglia, and the posterior parietal cortex (Arnsten and Li, 2005).

Along with the PFC, the caudate nucleus and its associated circuitry play an important role in the pathogenesis of ADHD. With MRI studies, structural abnormalities have been found in individuals with ADHD, related to reduced volumes of the left caudate nucleus and right anterior frontal cortex, indicating a reversal of the normal asymmetrical pattern. A bilateral reduction in the size of the putamen has also been found, as well as a reduction in the volume of the right globus pallidus (Cortese et al., 2012).

**FIGURE 3:**
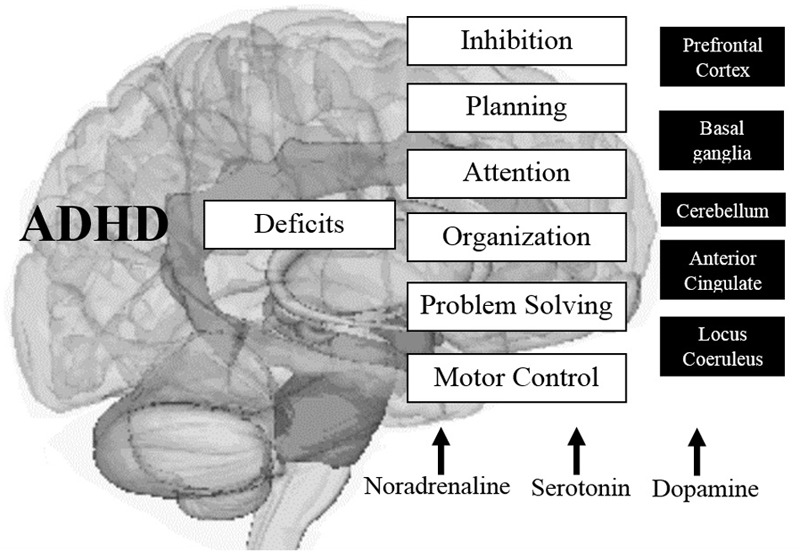
**Main altered brain structures in ADHD and their effects on cognitive components associated with EFs.** Source: made by myself.

It is also known that through the use of neuroimaging techniques, structures such as the cerebellum show significantly lower measurements in children with ADHD (on average) throughout childhood and adolescence, compared with the same structures in children without the disorder; In addition, the right PFC is slightly larger in the general population, and its counterpart in the left hemisphere is more symmetrical in people with the disorder, affecting mental abilities such as inhibition of responses, planning behavior, selective focus and organization of information necessary for solving problems and using specific cognitive operations (memory, metacognition, learning, and reasoning; Doyle, 2006; Cortese et al., 2012). See **Figure 3**.

Studies using functional neuroimaging techniques with high spatial [single-photon emission computed tomography (SPECT), PET, and fMRI] temporal [event-related brain potentials (ERPs)] and spatiotemporal [magnetoencephalography (MEG)] resolutions show functional differences in the PFC and striatum in patients with ADHD compared to controls, indicating the existence of dysfunction in the frontostriatal network, which could explain the changes observed in processes such as response inhibition. Furthermore, they showed a decrease in gray matter in the frontal right turn and the rotation of the right posterior cingulate, and in the middle left white matter (Cortese et al., 2012).

Electroencephalogram studies show the existence of a relationship between clinical symptoms of ADHD and characteristics of brain activity, reporting the presence of increased theta activity in the EEG (Boutros et al., 2005).

Volkow et al. (2011) has also investigated the biochemical implications of ADHD, determining that the neurobiological basis of the disorder presents a dysfunction of dopaminergic, serotonergic and noradrenergic circuits, causing an alteration in some cognitive mechanisms. Castellanos (1997) proposed the unitary theory of dopamine transmission in ADHD, based on abnormalities in two dopaminergic regions: (a) hypoactivation cortical regions (anterior cingulate), which produces a cognitive deficit and (b) overactivity in subcortical regions (caudate nucleus), causing excessive motor levels.

Arnsten et al. (1996) suggested that there may be different abnormalities in two noradrenergic regions: an underactive cortical (dorsolateral prefrontal), which referred to primary care deficits (WM) and overactivity in the subcortical systems (locus coeruleus), resulting in over-alertness.

The genetic mechanisms of ADHD are polygenic and this study focused on those related to dopamine, a gene involved in ADHD that could be the D2 receptor gene located on chromosome 11 (11q22-23; Comings, 2001). Other associated genes include the transporter gene norepinephrine (NET1) and the dopamine receptor gene D1 (DRD1; Bobb et al., 2005).

In general, the various neurobiological implications of ADHD can explain and help to understand the effects that are mainly exhibited in cognitive processes, such as the EFs.

### Inhibitory Control in Attention Deficit Disorder/Hyperactivity

One of the EFs most commonly affected in ADHD is inhibitory control (Doyle, 2006; Lange et al., 2010) and/or inability to inhibit responses associated with distracting stimuli (Arnsten and Li, 2005; Doyle, 2006).

Barkley (2006) argues that problems sustaining attention, such as ADHD, are the result of hypoactivity of the behavioral inhibition system, especially for poor interference control. He proposes a model (Barkley, 1997) in which he explains that the deficit in behavioral inhibition involves a delay or impairment in the development of four neuropsychological functions: the memory of non-verbal work, verbal WM, the self-regulation of emotion/motivation/activation, and reconstitution. The most important component of this model is the inhibition of behavior, which provides the basis for the neuropsychological skills mentioned; the other component of the model is the motor, which relates directly to the previous component and is mediated by the four EFs that control behavior.

Accordingly, Nigg (2001) shows clear evidence of the existence of a deficit in ADHD of different forms of executive inhibitory responses, but raises some doubts and questions about whether ADHD would be caused by a primary or secondary inhibitory disorder. To Nigg, although studies are consistent in the presence of an impulsive ADHD and disturbed behavior, the concept of inhibition should be refined, distinguishing between dependent inhibition of executive control and dependent inhibition of motivational Control; in turn, it suggests that the association between domains and deficits in ADHD is more related to inhibition on the strength of a major boost.

Attention deficit hyperactivity disorder is indeed associated with poor cognitive control, particularly inhibitory control (Willcutt et al., 2005; Chamberlain et al., 2011), this could explain why people with ADHD have slower RTs, suggesting the use of more time to inhibit prepotent responses in this population (Shen et al., 2011).

The inhibitory deficit in ADHD is associated with both structural and functional abnormalities in the frontostriatal and frontoparietal circuits, often revealing hypoactivation in prefrontal type tasks for go/no go, compared to the typical population (De La Fuente et al., 2013; Hart et al., 2013). Hervey et al. (2006) and Tamm et al. (2012) suggest that ADHD symptoms include poor performance on neuropsychological measures of inhibition, as well as associated measures (lack of foresight, lack of insight, difficulty delaying gratification, poor organization, poor sense of time, excessive responses).

Various studies of children with ADHD have identified alterations in sustained attention and inhibitory control, which leads to poor self-regulation and behavioral difficulties (Puentes-Rozo et al., 2008). These findings support the theory of the involvement of multioperational location systems in ADHD, including prefrontal and posterior cortical, reticular thalamic, striatal, limbic and mediated connections by specific neurotransmitter systems (Barkley, 2006).

Based on the above, the lack of inhibitory control in ADHD should not only be related to behavioral and cognitive impairments, but also to emotional disturbances, and although the deficit in emotional regulation is not currently one of the symptoms of ADHD diagnoses, various theoretical proposals suggest that it is a fundamental aspect of the disorder (Barkley, 2006).

Individuals with ADHD often have symptoms such as irritability, moodiness, low tolerance to frustration, and sudden and unpredictable changes associated with negative emotions such as anger and sadness (Sobanski et al., 2010), which could be explained by a deficit in emotional regulation, due to alterations in inhibitory control, as demonstrated by several studies which documented that individuals with ADHD not only suffer from attention difficulties, disorganization, hyperactivity and impulsivity, but also various emotional problems, such as emotional liability, excessive emotional reactivity, and irritability (Sobanski et al., 2010; Schoemaker et al., 2012; Arnett et al., 2013).

To Barkley (2006), emotional self-regulation is defined as a set of business processes that enable modular emotions and, upon receipt of a dysfunction, can lead to increased emotional responses to certain situations, as well as less empathy and less ability to regulate emotional states, hence the difficulties that individuals with ADHD have expressing emotions, a result of a primary dysfunction in the inhibitory control processes.

## Post-Traumatic Stress Disorder

### Conceptualization, Prevalence, Comorbidity, and Clinical Criteria

According to the APA and the guidance provided in the DSM-5 (American Psychiatric Association [APA], 2013), PTSD is characterized as a psychiatric disorder, described as a categorical entity clinical order. It is currently classified as one of the disorders related to trauma and stress factors, and can occur in people who have experienced or witnessed a traumatic event such as a natural disaster, a serious accident, an act of terrorism, war or combat, violent personal assault or rape. PTSD is part of a real disorder that leads to short and long term involvement after having experienced or witnessed an event of great emotional impact (Bryant et al., 2011; Sard, 2011). Psychological distress following exposure to a traumatic or stressful events is variable. It has been shown that some people who have been exposed to a stressful or traumatic event do not exhibit clinical features related to PTSD or pathogenesis. This reveals a major contribution by modern science in relation to the existing biological predisposition in some individuals associated with a low susceptibility to this disorder, and therefore provides arguments that PTSD represents a specific phenotype associated with a failure to recover from the normal effects of trauma (Yehuda and LeDoux, 2007).

Regarding the prevalence of PTSD, the data tends to vary according to the diagnostic criteria used to define the disorder, the evaluation procedures implemented, the characteristics of the sample and the context in which the event occurred. All these factors must be considered when studying the phenomenon (Doctor et al., 2011). In the USA, the lifetime risk for PTSD, using the DSM-IV (American Psychiatric Association [APA], 1994) criteria, at the age of 75 years was 8.7% (American Psychiatric Association [APA], 2013). The annual prevalence among USA adults is about 3.5% (Kessler and Wang, 2008). The estimates reported in Europe and most of Asia, Africa and Latin America are lower, at a volume of 0.5–1.0% (Kessler et al., 2005). Although, different groups show different levels of exposure to traumatic events, the conditional probability for PTSD may also vary between different cultural groups when they develop a similar level of exposure. The PTSD rates are higher among veterans and other people whose profession presents a high risk of traumatic exposure (e.g., police, firefighters, emergency medical personnel). The highest rates (ranging from a third to more than half of those exposed) are among the survivors of rape, military combat, captivity and confinement and political or ethnic genocide (Chapman and Diaz-Arrastia, 2014). Classic reference studies have shown that traumatic events with a greater probability of causing a disorder for both men and women are: rape, war, aggression or witness of a murder, and suffering physical abuse in the early stages of life (Kessler et al., 2005; Ogle et al., 2013).

Individuals with PTSD are 80% more likely to have symptoms that meet diagnostic criteria for at least one other mental disorder (e.g., depression, bipolar disorder, anxiety or substance abuse disorders; de Jong et al., 2001; American Psychiatric Association [APA], 2013). Studies with military personnel and veterans reveal a joint incidence of PTSD and mild traumatic brain injury for almost 48% of cases (Chapman and Diaz-Arrastia, 2014). Similarly, there is considerable comorbidity between PTSD and a major neurocognitive disorder (Doctor et al., 2011; Maksimovskiy et al., 2014; Pagotto et al., 2015), including a high association between PTSD and a significant decline in physical and mental health (Blakeley and Jansen, 2013; Pacella et al., 2013). The essential feature for the start of a PTSD diagnosis is the appearance of symptoms after exposure to one or more traumatic events, either directly or indirectly experienced, such as witnessing something experienced by others. According to the DSM-5 (American Psychiatric Association [APA], 2013) criteria are applicable for adults, adolescents and children over 6 years of age, such as the presence of symptoms persisting for more than a month after experiencing the stressor event. The alteration leads to clinically significant distress or impairment in social, occupational or other important areas of functioning of the individual. The alteration cannot be attributed to the physiological effects of a substance (e.g., drugs, alcohol) or another medical condition (American Psychiatric Association [APA], 2013; Friedman, 2013).

Symptoms that form as a result of the traumatic experience are organized into categories: *Re-experiences or intrusions, Avoidance, Persistent negative alterations in ognition and mood*, and *Increased excitability* (Bryant et al., 2011; American Psychiatric Association [APA], 2013). Currently, there are criteria for children under 6 years in relation to the diagnosis of PTSD, as well as indications of clinical order as to the late expression of the disorder (Friedman et al., 2011; American Psychiatric Association [APA], 2013). Psychiatric indications are clear as to their non-linearity in the symptomatic expression in PTSD, as their presentation is varied, occurring specifically in many cases and in others displaying a combination of symptoms (Friedman, 2013). According to the literature, during the development and course of PTSD the symptoms usually begin within the first 3 months after the trauma, although there may be a delay of months, or even years, before the diagnostic criteria are met, which has been called “delayed expression” disorder (Greenberg et al., 2015). It must also specify if the individual experiences persistent dissociative symptoms (Friedman et al., 2011; American Psychiatric Association [APA], 2013). See **Table 2**.

**Table 2:** Summary of specific criteria and symptoms for the diagnosis of PTSD (American Psychiatric Association [APA], 2013).

Re-experiences or intrusions (1/5)	
(1) Recurrent, involuntary, and intrusive distressing memories of the traumatic event(s).	(2) Recurrent distressing dreams in which the content and/or affect of the dream are related to the traumatic event(s).
(3) Dissociative reactions (e.g., flashbacks) in which the individual feels or acts as if the traumatic event(s) were recurring.	(4) Intense or prolonged psychological distress at exposure to internal or external cues that symbolize or resemble an aspect of the traumatic event(s).
(5) Marked physiological reactions to internal or external cues that symbolize or resemble an aspect of the traumatic event(s).	
**Avoidance (1/2)**	
(1) Avoidance or efforts to avoid distressing memories, thoughts or feelings about or closely associated with the traumatic event(s).	(2) Avoidance or efforts to avoid external reminders (people, places, conversations, activities, objects, situations) that arouse distressing memories, thoughts, or feelings about or closely associated with the traumatic event(s).
**Persistent negative alterations in cognition and mood (2/7)**	
(1) Inability to remember an important aspect of the traumatic event(s).	(2) Persistent and exaggerated negative beliefs or expectations about oneself, others, or the world.
(3) Persistent, distorted cognitions about the cause or consequences of the traumatic event(s) that lead the individual to blame himself/herself or others.	(4) Persistent negative emotional state.
(5) Markedly diminished interest or participation in significant activities.	(6) Feelings of detachment or estrangement from others.
(7) Persistent inability to experience positive emotions.	
**Increased excitability (2/6)**	
(1) Irritable behavior and angry outbursts (with little or no provocation) typically expressed as verbal or physical aggression toward people or objects.	(2) Reckless or self-destructive behavior.
(3) Hypervigilance.	(4) Exaggerated startle response.
(5) Problems with concentration.	(6) Sleep disturbance.

*Applies to adults, adolescents and children over 6 years. The essential feature (PTSD) is the development of symptoms after exposure to one or more traumatic events. The duration of symptoms of disturbance should be more than 1 month. Dissociative symptoms should not be attributed to the physiological effects of a substance. (/): symptoms required for the category. The disturbance causes clinically significant distress or impairment in important social, occupational, or other areas of functioning.*

### Biological Bases in Post-traumatic Stress Disorder

Irregularities in the biological system involved in the response to stressful events largely lead to damages in the body, setting a pathological mechanism, compared to normal behavior (Yehuda and LeDoux, 2007; Skelton et al., 2012). Dysfunctions in the spinal sympathetic-adrenal-axis and the hypothalamic-pituitary-adrenal-cortical axis, neuroendocrine dysregulation in glutamatergic, noradrenergic, and serotonin systems result in an imbalance and consequently an increased vulnerability to developing PTSD (Pitman et al., 2012).

Studies using models of psychological stress to evaluate the function of the HPA axis revealed an exaggerated cortisol response and hypersecretion of CRH in the PTSD-state, as a result of physiological failures of the system (Dedovic et al., 2009; Sherin and Nemeroff, 2011; Pitman et al., 2012). Paradoxically, there is evidence showing low plasma cortisol levels and an increase in the negative feedback of the HPA axis under basal conditions (Pitman et al., 2012). The presence of low concentrations of cortisol in people who survive traumatic events is a fact that seems contrary to the idea that stress may be associated with elevated levels of cortisol. One explanation that can be made is that in the course of adaptation to trauma, low cortisol concentrations are detected first, reflecting a chronic adaptation of the HPA axis to stress.

**FIGURE 4:**
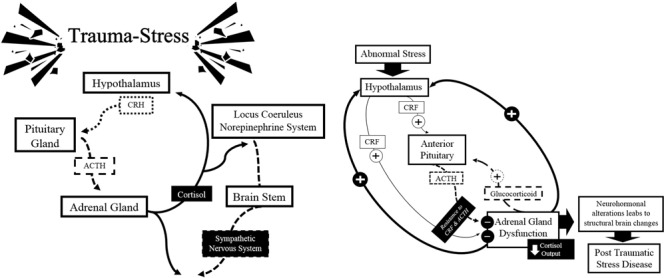
**Schematic summary of the classical model of neurobiology in the expression of PTSD: hypothalamic-pituitary-adrenal axis.** Source: made by myself.

Increased and persistent activation of CRH has also been associated with increased activity of the autonomic nervous system in PTSD victims, particularly in a group of women with a history of sexual abuse in childhood with symptoms of depression and anxiety (Heim et al., 2000). The increase in the negative feedback generated by the cortisol could offer an explanation to why the concentration of cortisol is low in the presence of elevated levels of CRH. Yehuda and LeDoux (2007) propose that chronic release of CRH produces an abnormal response by the pituitary gland. Since the number and sensitivity of lymphocytic glucocorticoid receptors is increased, the negative feedback is also increased, causing an attenuation of the cortisol. This increased negative feedback contrasts with the drop that occurs in depression, in which the chronic release of CRH causes decreased negative feedback leading to hypercortisolism and down-regulation of glucocorticoid receptors (Sherin and Nemeroff, 2011; Pitman et al., 2012). See **Figure 4**.

The hypothesis, which postulates that it exists because of chronic stress on the system, is based on the principle known as down-regulation of the GABAergic system, which leads to an excessive and constant activation of glutamate, which can induce long-term synaptic changes and therefore can damage the cognitive and emotional order. Equally important is the inhibition of GABAergic anomalies associated with cellular toxicity, which leads to high concentrations of harmful cell agents, and therefore a decrease in neuroplasticity, and in extreme cases, their death (Hasler et al., 2010; Sherin and Nemeroff, 2011; Pitman et al., 2012). Biochemical studies also reported a central noradrenergic dysfunction, associated with psychiatric symptoms of PTSD, indicating, in some cases, a noradrenergic hypersensitivity and a possible down-regulation of neural receptors that affect metabolic activity (Sherin and Nemeroff, 2011; Pitman et al., 2012; Yamamoto et al., 2013). The regulation of serotonin (5-HT) and consequently the release of corticosteroids under stressful events are positively associated with increased secretion of CRH and PTSD (Sherin and Nemeroff, 2011).

### Neural Circuitry Alterations after Traumatic Events

Changes of the biological order, in most cases, lead to structural and functional flaws in several neural circuits after traumatic events (Bremner et al., 2008). The repercussions are evident in the set of clinical symptoms of the disorder (Suvak and Barrett, 2011). Affectations of anatomical and functional natures have been identified in the neurocircuits, especially in the medial PFC, hippocampus, and amygdala (Shin et al., 2006; Herringa et al., 2012). Similarly, cortical regions such as the insular cortex, anterior cingulate cortex, thalamus, and subcortical limbic structures have become important in understanding the anomalies present in PTSD (Herringa et al., 2012). Although, there is a high reproducibility in terms of deficits in classical structures of the human nervous system, there are also novel reports in the scientific literature that reveal the complexity of the neurobiological mechanisms involved in the expression and development of psychopathology in PTSD (Herringa et al., 2012; Patel et al., 2012). There is no equality regarding variables such as study design, type of target population, biological technique, time of occurrence of the disorder, the expression of symptoms and its comorbidities with others, leading to absolute certainty of one single structure and a single key fault disorder (Etkin and Wager, 2007; Pitman et al., 2012).

The volume reduction in the hippocampus and hippocampal atrophy phenomenon detected in patients with chronic PTSD has been widely reported (Shin et al., 2006; Pitman et al., 2012). The most widespread hypothesis corresponds to a phenomenon of cytotoxicity and cell death, resulting from excessive stimulation of the system (Pitman et al., 2012). A decrease in hippocampal activation during symptomatic expression has also been reported, as well as in performing memory tasks involving a judgment of emotional valences (Sherin and Nemeroff, 2011). Conversely, changes in the system (5-HT) reach defined alterations in hippocampal increased activity and severity of symptoms in PTSD (Bremner, 2006; Bremner et al., 2008; Sherin and Nemeroff, 2011).

Amygdala deficits lead to flaws in the evaluation and regulation of biologically relevant threat signals. There is evidence of abnormalities in the functionality of the amygdala in PTSD, and increased startle response has been postulated to represent an increase in function (Bremner, 2006). Because of this, a large brain circuitry is involved in their functional ability, which is consequently committed to the ventromedial PFC and rostral anterior cingulate region (Sherin and Nemeroff, 2011; Pitman et al., 2012). Similarly, chronic PTSD has been correlated with a reduction in the dorsolateral prefrontal parietal gray matter and cingulate cortex (Pitman et al., 2012). The duration of the disorder has been analyzed as a direct proportion to the reduction in gray matter and the severity of the symptoms (Eckart et al., 2011; Chen et al., 2012; Pitman et al., 2012). Effects on the brain’s medial diencephalic region, specifically the thalamus, by glutamatergic system failures have been associated with an expression of dissociative states in PTSD (Bremner et al., 2008). It is speculated that such dissociation occurs as a result of alterations in the thalamic transmission, which can be due either to a decrease in entries in the thalamus, which generates a suppression of emotional memories, or charging excessive noradrenergic input stimuli in the thalamus (O’Brien and Nutt, 1998).

Sartory et al. (2013) recently demonstrated a greater number of interconnected cortical areas involved in shaping the symptoms, specifically re-experiencing on a recurring basis. PTSD patients showed significant activation in the mid-line retrosplenial cortex and precuneus region in response to stimuli associated with the trauma. Likewise, a hyperarousal of the anterior cingulate gyrus was also evident as well as the bilateral amygdala and hypoactivation in sensory association areas, which could indicate that the attentional system required to meet the demand of a task is compromised by centralization of a traumatic memory, generating an associative learning bias due to its intrusive and disturbing connotations.

It is important to note that although research reveals a variety of structural and functional changes by neuroimaging evidence due to exposure to a traumatic event, these changes have been associated with the trauma and therefore care should be taken not to consider them as a cause-effect (Sherin and Nemeroff, 2011; Patel et al., 2012).

Genetic vulnerability, as well as the pre-existence of different abnormalities, are aspects that could be acting as a development risk factor of a symptomatic trauma or neurodegenerative pattern after the trauma (Miller and Sadeh, 2014). Knowledge of the genetic basis and neural pathways involved in the etiology and maintenance of the disorder allow better understanding from a clinical perspective (Suvak and Barrett, 2011; Pitman et al., 2012; Skelton et al., 2012).

### Post-traumatic Stress Disorder and Inhibitory Control Dysfunction

According to the DSM-5 (American Psychiatric Association [APA], 2013), recurring memories of the event, avoidance of stimuli associated with the traumatic experience, diminished interest in significant activities, a constant state of hypervigilance, and attentional problems in terms of maintenance, form a pattern of information processing for the majority of PTSD cases.

The central components refer to neurobiological substrate irregularities involving an increased vulnerability in limbic and prefrontal regions, specifically during mnemonic, attentional, emotional and executive processing (Bonelli and Cummings, 2007; Pitman et al., 2012; Chen and Etkin, 2013).

Irregular functioning of inhibitory control, considering the properties of control and regulation, would lead to a permanent imbalance of the nervous system, as well as recurrent failures that would place the cognitive and emotional stability of the subject at risk (MacDonald et al., 2000; Gifford, 2002). The integrity of all executive capabilities provides benefits in terms of the strategy to direct attention and inhibit irrelevant stimuli, thus tending to best guarantee the achievement of a goal (Snyder et al., 2015). Failures in attentional efficiency of the executive control network, mainly in inhibiting interference and attentional failures, have been linked to a decreased ability to control voluntary actions in PTSD patients (Pacheco-Unguetti et al., 2011; Pechtel and Pizzagalli, 2011), as well as difficulties in developing efficient strategies to cope with potentially anxiety-inducing elements (Polak et al., 2012).

Inhibitory control is essential for the exercise of mental flexibility, control and impulsivity interference, WM, self-regulation of emotion and the capacity for analysis and synthesis of behavior (Aron, 2007; Diamond, 2013). An extensive network of frontostriatal circuits are affected in the inhibitory control of patients who have suffered traumatic experiences (Aupperle et al., 2012b; Pitman et al., 2012). Neuropsychological studies have documented anatomical and functional disorders associated with cognitive deficits in patients with PTSD, compared with controls (Bremner, 2006). Long-term effects have been identified in the memory domain for verbal information (McNally, 2006; Brewin, 2011) and visual memory (Marx et al., 2009), declarative memory (Samuelson, 2011), attention (Samuelson et al., 2006; Aupperle et al., 2012a), sustained attention (Vasterling et al., 2002), emotional processing (Milad et al., 2008), WM (Aupperle et al., 2012a), intelligence (Gilbertson et al., 2006), language and communication (McNally, 2006), learning (Samuelson et al., 2006), and processing speed (Samuelson et al., 2006). Disturbances in executive functioning are important to the large bias in cognitive activity in general (Pollak et al., 2010; Aupperle et al., 2012b).

### Relationship between Attention Deficit Disorder/Hyperactivity and Post-traumatic Stress Disorder

The inhibitory control component of interest in this review lies mainly in its importance as a central aspect of EF (Banich, 2009; Diamond, 2013) and as a key mechanism that contributes significantly to the efficiency of the executive system in general (Aron, 2007; Banich, 2009). It is for this reason that the different components that make up the theory associated with EFs are initially highlighted, with special emphasis on inhibitory control.

The structural and functional abnormalities of both ADHD and PTSD are evident in a large and complex brain circuit that comprises frontal and medial areas, as well as the cingulate cortex and thalamus, the hippocampal formation and the amygdaloid complex (Bonelli and Cummings, 2007; Pitman et al., 2012; Etkin et al., 2013). This creates a permanent interest in their revision, seeking greater clarity of their underlying mechanisms, which can be a possible route in terms of objective biomarkers and its future use in the monitoring of each pathology.

On the other hand, endocrine factors and brain circuits are involved in the optimal functioning of inhibitory control, especially in the dorsal and ventral PFC, the supplementary motor area, anterior cingulate and parietal and occipital lobe. Such irregularities in the neural mechanisms are associated with the severity of the symptoms in both ADHD and PTSD, referring globally to deficits in attentional systems (Gifford, 2002; Rae et al., 2015).

Regarding the link between these two diagnostic entities, there is evidence suggesting comorbidity with prevalence estimates ranging from 12 to 37% (Adler et al., 2004). The comorbidity described is associated with problems in emotional modulation (arousal levels) that are common to both disorders (hyperarousal and hypoactivation; Harrington et al., 2012).

Adler et al. (2004) suggest that ADHD can become a risk factor that increases vulnerability to developing PTSD after exposure to a traumatic event, based on the finding that the patients with PTSD that they evaluated reported higher levels of ADHD in childhood compared to patients with other disorders.

A study by Koenen et al. (2007) showed patients were 50% more likely to experience psychological trauma if there was a history associated with hyperactivity problems, antisocial behavior, and irritability during childhood, compared to individuals with no such history.

Authors such as Adams (2010) have argued that experiencing traumatic events in childhood is strongly associated with development of psychiatric conditions for life, such as personality disorders, behavioral disorders, ADHD, depression, anxiety, substance abuse and PTSD; as well as developmental delay, impaired cognitive skills, learning difficulties, and even a lower IQ.

In 2000, Ford and collaborators revealed results that suggest that the presence of ADHD and Oppositional Defiant Disorder is more common in patients with a history of physical or sexual abuse, pointing to abuse as a significant risk factor for development of behavioral disorders.

Thus, ADHD during childhood increases exposure to trauma, including physical injuries, physical and sexual abuse, neglect, among others (Koenen et al., 2007; Ouyang et al., 2008). In addition, children who are exposed to traumatic events may be more vulnerable to experiencing an exacerbation of the symptoms of ADHD, such as those related to the regulation of impulses and physiological hyperarousal (Ford et al., 2000; Harrington et al., 2012).

The symptoms that reflect the overlap between ADHD and PTSD include irritability, excessive motor activity, learning disabilities, attention, the attentional shift and concentration difficulties, impulsive behavior, and exaggerated startle responses (Hervey et al., 2004; Daud and Rydelius, 2009), clearly demonstrating symptoms common to both disorders associated with EFs. Thus, alterations in the EFs due to ADHD may explain the high reporting of the disorder as a risk factor for developing PTSD, as suggested by several studies (Nigg et al., 2002; Martel and Nigg, 2006; Volkow et al., 2011).

Generally, according to Bernardi et al. (2012), high rates of impulsive behavior, inattentiveness, and alterations in inhibitory control in individuals with ADHD could provide explanations for the increased risk of trauma.

According to a study by Harrington et al. (2012), which used a confirmatory factor analysis to establish the level of correlation between ADHD and PTSD, the “lack of attention” in ADHD was identified as a factor associated moderately with symptoms of avoidance in the PTSD. Based on this result, the study authors explain that inattention in ADHD and avoidance symptoms in PTSD may reflect similar changes in cognitive control mechanisms, creating problems of distraction and disorganization in ADHD and difficulty in emotionally suppressing intrusive thoughts in PTSD, exposing possible associations between the two disorders related to alterations in inhibitory control.

Swick et al. (2013) evaluated the variability in RT as an indicator of executive dysfunction, specifically as it relates to difficulties in inhibitory control and excessive mental disorder in war veterans and controls, through the go/no go inhibition task, finding that individuals with PTSD had significantly greater variability in RT than the controls, suggesting that they are less successful in inhibiting appropriate responses; in addition, other symptoms such as attentional impulsivity are also present in individuals with ADHD. The findings of this study pointed to deficits in cognitive control processes, specifically top–down, which could contribute to the continuation of PTSD symptoms.

Greater RT variability in cognitive tasks has been associated with a greater propensity toward negative affect (Ode et al., 2011), among other conditions, for developmental disorders (Tamm et al., 2012) such as ADHD. In fact, the increased variability in RTs in ADHD is a highly replicable finding (Hervey et al., 2006; Swick et al., 2013; Tarantino et al., 2013). Among the various explanations for this increased RT variability are: deficits in sustained attention, problems processing time information, and difficulties in regulating behavior (Johnson et al., 2007; Tamm et al., 2012), resulting in a general marker of executive dysfunction (Ode et al., 2011).

However, it is not known whether deficiencies in the general cognitive mechanism such as top–down can account for similar deficits in several disorders, or whether different mechanisms are involved (Swick et al., 2013).

Based on the above, it could be argued that the cognitive, emotional and behavioral disfunctions that characterize both ADHD and PTSD may result from the overlapping of neural substrates.

## General Conclusion

As a result of the review of literature associated with ADHD and PTSD, it can be said that these two clinical entities are characterized by a set of signs and symptoms that detail their particular neurobiology, thus allowing a better understanding for clinical management and behavioral aspects to be taken into account in the different diagnoses and future interventions.

Inhibitory control is a cognitive process that plays an important role in ADHD and PTSD; giving rise to the possibility that research generates experimental approaches where inhibitory control is associated with emotional components. In this regard, it would be possible to act on the “psychological distress” that leads these two disorders, especially regarding behavioral disturbances, threatening the adaptation of the affected subject to different contexts in which they operate, including comorbidity with various other mental disorders (Bonelli and Cummings, 2007; Etkin et al., 2013).

Current information on the link between ADHD and PTSD is inconclusive, which still allows for the development of knowledge and interest in its disclosure, primarily through the generation of new research.

On the other hand, continuing to generate associated research will undoubtedly provide a greater understanding as to the specificity of the neurobiological aspects present in both psychiatric conditions. In this sense, the following may be proposed: (1) Biomarker analysis and its future use in the monitoring of each pathology. Genetic and neurophysiological evidence can be integrated into the study of brain hemodynamics using fMRI, PET and SPECT techniques, for example, as well as EEG by monitoring spontaneous and induced electrical activity. Thus, we could better understand the physiological and cognitive mechanisms underlying these pathologic entities, generating new possibilities for clinical and experimental settings. (2) Use of neuropsychological assessment and neuroimaging in order to map the brain. It is vital to develop studies that record biological activity while simultaneously making assessments via cognitive tests (specifically frontal lobe assessment using EF tests). It would be more appropriate to combine traditional and ecological neuropsychological tools in order to obtain more reliable results. (3) Comorbidity and monitoring of the disorder. Given the explanations outlined in this review regarding the increased chance of individuals diagnosed with ADHD suffering from PTSD, is it possible to argue that ADHD worsens the outcome of PTSD, and can we therefore propose a comorbidity? If yes, this could be included in future diagnostic manuals to show the interaction between the two pathologies. Thus, it would contribute not only to the understanding and diagnosis of both diseases, but also to its monitoring at both the clinical and research levels.

Finally, it should be expected that the inclusion of technological tools in the diagnostic and intervention stage will allow better recreation of the scenarios of daily life, thus increasing understanding of rarely addressed cognitive and emotional strategies that are important in shaping ADHD and PTSD.

## Author Contributions

LM, EP, CS, MT, CT conceptualized and designed the work. LM, EP, CS drafted the work. LM, EP, CS, MT, CT critically revised and approved the manuscript.

## Conflict of Interest Statement

The authors declare that the research was conducted in the absence of any commercial or financial relationships that could be construed as a potential conflict of interest.
